# Evidence that an intervention weakens the relationship between adolescent electronic cigarette use and tobacco smoking: a 24-month prospective study

**DOI:** 10.1136/tobaccocontrol-2018-054905

**Published:** 2019-06-28

**Authors:** Mark Conner, Sarah Grogan, Ruth Simms-Ellis, Keira Flett, Bianca Sykes-Muskett, Lisa Cowap, Rebecca Lawton, Christopher Armitage, David Meads, Laetitia Schmitt, Carole Torgerson, Robert West, Kamran Siddiqi

**Affiliations:** 1 School of Psychology, University of Leeds, Leeds, UK; 2 Department of Psychology, Manchester Metropolitan University, Manchester, UK; 3 Faculty of Health Sciences, Staffordshire University, Staffordshire, UK; 4 Psychology, University of Manchester, Manchester, Greater Manchester, UK; 5 Institute of Health Sciences, University of Leeds, Leeds, UK; 6 Academic Centre for Health Economics, University of York, York, UK; 7 Education, Durham University, Durham, Durham, UK; 8 Institute of Health Sciences, University of York, York, North Yorkshire, UK

**Keywords:** electronic nicotine delivery systems, e-cigarettes, smoking, harm reduction, intervention

## Abstract

**Background:**

The electronic cigarette (e-cigarette) use to subsequent smoking relationship in adolescents has received much attention. Whether an intervention to reduce smoking initiation attenuated this relationship was assessed.

**Method:**

Data were from 3994 adolescent never smokers (aged 13–14 years at baseline) as part of a cluster randomised controlled trial. Self-report measures of smoking, e-cigarette use and covariates were assessed and used to predict ever smoked cigarettes, any recent tobacco smoking and regularly smoked cigarettes at 24-month follow-up.

**Results:**

Baseline ever use of e-cigarettes was associated with ever smoked cigarettes (OR=4.03, 95% CI 3.33 to 4.88; controlling for covariates, OR=2.78, 95% CI 2.20 to 3.51), any recent tobacco smoking (OR=3.38, 95% CI 2.72 to 4.21; controlling for covariates, OR=2.17, 95% CI 1.76 to 2.69) and regularly smoked cigarettes (OR=3.60, 95% CI 2.35 to 5.51; controlling for covariates, OR=1.27, 95% CI 1.17 to 1.39) at follow-up. For ever smoked cigarettes only, the impact of e-cigarette use was attenuated in the intervention (OR=1.83) compared with control (OR=4.53) condition. For ever smoked cigarettes and any recent tobacco smoking, the impact of e-cigarette use was attenuated among those with friends who smoked (OR=2.05 (ever smoked); 1·53 (any tobacco use)) compared with those without friends who smoked (OR=3.32 (ever smoked); 2·17 (any tobacco use)).

**Conclusions:**

This is one of the first studies to show that e-cigarette use was robustly associated with measures of smoking over 24 months and the first to show an intervention to attenuate the relationship. Further research with a broader age range of adolescents is required.

## Introduction

The potential beneficial impact of electronic cigarettes (e-cigarettes) on quitting cigarette smoking has been identified.^1–4^ At the same time, the increasing use of e-cigarettes in adolescents (particularly in those who do not smoke) has raised concerns. A focus of attention has been on the role of e-cigarettes in smoking initiation. Recent cross-sectional surveys of cohorts in both the USA^5–8^ and UK^9–11^ have shown that adolescents’ rates of ever use of e-cigarettes increased at the same time that rates of cigarette use decreased. In contrast, longitudinal surveys in adolescents show use of e-cigarettes to be associated with subsequent smoking initiation. For example, US studies show e-cigarette use to be positively associated with initiation of smoking 12–24 months later in 14–16-year olds.^12–15^ Similar results have been reported in UK studies^16 17^ of 14-year olds over the periods of 12 months.

The present research reports post-hoc analysis of a subset of data from a cluster randomised controlled trial of an intervention on reducing smoking in 16-year olds.^18 19^ This intervention significantly reduced ever smoking and any recent tobacco use but had mixed effects on regular smoking and breath carbon monoxide.^19^ Here, we analysed data from adolescents who were never smokers and either ever or never users of e-cigarettes at age 14. The focus was on predicting smoking at age 16 (24 months later). A previous paper reported the impact of e-cigarette use at age 14 among never smokers on smoking at age 15 (12 months later) in the control condition.^16^ The present study aimed to extend knowledge in this area in four important ways. First, this is the first study to examine the effects of an intervention designed to reduce adolescent smoking initiation on relationships between e-cigarette use and subsequent smoking. Second, this study explored associations over longer periods of time, doubling the most commonly reported follow-up period of 12 months (24 months, age 14 at baseline, age 16 at follow-up). Third, this study assessed the impact beyond any smoking postbaseline to examine any recent tobacco smoking and regular smoking at follow-up (only Barrington-Trimis *et al* 15 report data on regular smoking). Fourth, self-reported measures of smoking at follow-up were validated against objective smoking measures (see^16^ for similar approach).

In summary, the present research assessed relationships between e-cigarette use and smoking (objectively validated) 24 months later in a sample of UK adolescents aged 14 at baseline. Moderating effects of an intervention and effects of controlling for a number of covariates (gender, ethnicity, individual/school level socioeconomic status, friends and family smoking, impulsivity and intentions, attitudes, norms, perceived behavioural control and self-efficacy in relation to smoking) were also assessed. No previous study has examined the impact of an intervention designed to reduce smoking initiation^18 19^ on relationships between e-cigarette use and subsequent smoking. The moderating effect of friends smoking was also explored. Two previous UK studies^16 17^ had shown the impact of e-cigarettes on subsequent smoking initiation to be significantly stronger in those with no friends who smoked compared those with friends who smoke.

## Methods

### Participants and procedures

Data were collected as part of a preregistered, 4-year cluster randomised controlled trial (RCT) of a school-based intervention to prevent smoking initiation^18 19^ using implementation intentions.^20–22^ The study was conducted in 45 schools in England with adolescents initially aged 11–12 years. Those in intervention schools (coded 1) read antismoking messages and formed implementation intentions about how to refuse offers of cigarettes on eight occasions (twice per year), for example, “If offered a cigarette, I will say… no cancer sticks for me”. Those in control schools (coded 0) formed implementation intentions in relation to completing homework. Head teachers consented to school participation with parents given option to withdraw children from the study. Adolescents consented by completing questionnaires matched across time points by code. The cluster RCT^19^ showed that formation of implementation intentions in relation to cigarette offers significantly reduced ever smoking cigarettes and any combustible tobacco use in the last 30 days but not regular cigarette smoking or breath carbon monoxide levels at follow-up. The data reported here are from waves 3 (September–December 2014; referred to as baseline in 13–14-year olds) and 5 (September–December 2016; referred to as follow-up in 15–16-year olds) of the trial when e-cigarette use measures were added to the data collection. Only respondents reporting having never smoked a cigarette at baseline were included here (ie, this is a post-hoc analysis). A previous study reported the impact of e-cigarette use (wave 3) on smoking 12 months later (wave 4) in the control condition.^16^

The University of Leeds, UK (Faculty of Medicine) ethical review committee approved the study (reference 12–0155).

### Measures

Cigarette use was assessed using standardised measures^23^ at both time points; adolescents ticked one of: ‘I have never smoked; I have only tried smoking once; I used to smoke sometimes, but I never smoke cigarettes now; I sometimes smoke cigarettes now, but I don’t smoke as many as one a week; I usually smoke between one and six cigarettes a week and I usually smoke more than six cigarettes a week’. Only respondents marking the first response at baseline were retained for analysis. At follow-up, this measure was converted into a measure of ever smoked cigarettes (first response coded 0; other responses coded 1) and regularly smoked cigarettes (last two responses coded 1; other responses coded 0). At follow-up only, respondents indicated on how many days in the last 30 they had used (tobacco) cigarettes, cigars, pipes and sheesha (flavoured tobacco); converted to a measure of any recent tobacco smoking (no days smoking=0; one or more days smoking=1). The self-reported smoking measures at follow-up were validated against a measure of breath carbon monoxide (CO) levels (Micro+Smokerlyzer CO Monitor, Bedfont Scientific Limited, Kent, England), although we did not reclassify self-report measures based on CO level. Such measures are reliable and valid ways of assessing regular cigarette smoking^24 25^ but not occasional smoking due to the short half-life (4–6 hours) of breath CO.

E-cigarettes/vapourisers were described as ‘a tube that sometimes looks like a normal cigarette and has a glowing tip. They all puff a vapour that looks like smoke but unlike normal cigarettes, they don’t burn tobacco’. Use of e-cigarettes at baseline was tapped by one item (‘Which ONE of the following is closest to describing your experience of e-cigarettes or vapourisers’, I have never used them; I have tried them once or twice; I use them sometimes (more than once a month but less than once a week); I use them often (more than once a week)’), converted into a measure of ever used e-cigarettes (first response=0; other responses=1).

Other measures were assessed as covariates/moderators. Demographic variables included gender (boy=0; girl=1), ethnicity (non-white=0, white=1), family affluence (based on the 4-item Family Affluence scale^26^: scored 0–3, higher scores indicating greater affluence), percentage of children per school eligible for free school meals (<median 20%=0; >20%=1).^27^ Friends’ smoking was assessed using the question, ‘How many of your friends smoke?’, none of them; only a few; half and half; most but not all; all of them (none of them=0; a few or more=1). Family smoking at baseline was assessed using the question: ‘Who smokes in your family now? Tick all the people who smoke at the moment’, followed by a list of family members (no family members=0; one or more family members=1). Impulsiveness was measured at follow-up based on a 5-item impulsivity scale (scored 0–5, higher scores indicating greater impulsivity).^28^

Health cognitions about smoking^21^ were assessed as mean of multiple questions on 5-point scales: intention (three questions; for example, ‘I plan not to smoke’; Cronbach’s α=0·90); attitude (seven questions; for example, ‘For me, smoking would be… good-bad’; α=0·87); perceived norms (five questions; for example, ‘Most of my friends think… I should smoke–I should not smoke’; α=0·79); perceived behavioural control (three questions; for example, ‘I am confident I could resist smoking’, strongly disagree–strongly agree; α=0·69); self-efficacy (six questions; for example, ‘I can say no to smoking, even at school’ strongly disagree–strongly agree; α=0·91). Questions were highly skewed towards negative views of smoking and so were dichotomised (negative views=0; less negative views=1). Based on previous findings,^16 17^ an interaction between e-cigarette use and friends smoking was also computed.

### Data analysis

Missing data ranged from 0.0% (gender) to 1.4% (ethnicity) and 96% of the 3994 never smokers in the sample would have been available for analysis under the traditional listwise deletion method across variables. Data were missing due to item non-response. As level of missing values was low, missing at random was assumed to justify multiple imputation. Multiple imputation in SPSS 24 generated five imputed datasets. Imputed values compared reasonably to observed values, and results using listwise deletion were similar to multiple imputation, so imputed results are presented. SPSS was used to analyse descriptives on all measures, to examine relationships between ever used e-cigarette at baseline and smoking measures at follow-up and validate smoking measures against follow-up breath CO levels (using logistic regressions).

The main analyses used hierarchical linear models HLM7^29^ to predict follow-up ever smoked cigarettes, any recent tobacco smoking or regularly smoked cigarettes based on ever use of e-cigarettes and covariates. Model 1 controlled for the clustering of adolescents within schools and used baseline ever use of e-cigarettes as a predictor. Model 2 added covariates, and model 3 tested interactions between condition and each covariate (cross-level interaction). All multilevel analyses used random slopes and population average model with robust SEs. Analyses were conducted for each of five imputed datasets, and the results combined using Rubin’s rules. For each predictor, we report the OR, 95% CI and p value. We also report the −2 log-likelihood to indicate model fit. When significant, we decomposed the interaction between e-cigarette use and friends smoking using the free software provided by Preacher (model 1; http://www.quantpsy.org/interact/hlm2.htm). For significant cross-level interactions between intervention condition and predictors of smoking, we compared the effects for predictors estimated separately in control and intervention conditions.

## Results

### Sample description

Table 1 provides descriptive data on all measures for the full imputed sample. At baseline, the sample of 3994 never smokers (table 1) comprised 47.7% males, with a majority (81.0%) not having ever used e-cigarettes. At follow-up, 24 months later, 20.3% had ever smoked cigarettes, 9.7% reported any recent tobacco smoking, while 2.1% regularly smoked cigarettes (table 1). At follow-up, breath CO levels were significantly higher in those reporting ever versus never having smoked cigarettes (p<0.001), in those reporting one or more versus zero days any recent tobacco smoking (p<0.001) and in regularly smoked cigarettes versus other groups (p<0.001).

**Table 1:** Descriptive data for the sample (n=3994)

	n (%)
Gender	
Male	1904 (47.7)
Female	2090 (52.3)
Ethnicity	
White	688 (17.2)
Non-white	3306 (82.8)
Family affluence1	2.72 (0.49)
Ever used e-cigarettes (baseline)	
No	3236 (81.0)
Yes	758 (19.0)
Friend smokers	
None	2602 (65.1)
A few or more	1392 (34.9)
Family smokers	
None	1502 (37.6)
One or more	2492 (62.4)
Impulsivity*	2.04 (1.65)
Intention	
Low	3624 (90.7)
High	370 (9.3)
Attitude	
Low	3302 (82.7)
High	692 (17.3)
Norms	
Low	3455 (86.5)
High	539 (13.5)
Perceived behavioural control	
Low	3260 (81.6)
High	734 (18.4)
Self-efficacy	
Low	3239 (81.1)
High	755 (18.9)
Free school meals†	
Low	22 (48.9)
High	23 (51.1)
Condition†	
Control	20 (44.4)
Intervention	25 (55.6)
Ever smoked cigarettes (follow-up)	
No	3180 (79.6)
Yes	814 (20.4)
Any recent tobacco smoking (follow-up)	
No	3607 (90.3)
Yes	387 (9.7)
Regularly smoked cigarettes (follow-up)	
No	3910 (97.9)
Yes	84 (2.1)

*Mean and SD for these variables.

†Number of schools.

### Simple relationships between baseline use of e-cigarettes and follow-up smoking

Table 2 shows the numbers reporting different levels of smoking cigarettes or any recent tobacco smoking at follow-up split by baseline ever used e-cigarette. Ever smoked cigarettes at follow-up was 15.2% (492/3235) for those not using versus 42.4% (322/759) for those ever using e-cigarettes at baseline. The figures for any recent tobacco smoking was 7.0% (226/3236) for those not using versus 21.2% (161/758) for those ever using e-cigarettes at baseline. Regularly smoked cigarettes at follow-up was 1.5% (47/3235) for those not using versus 4.9% (37/759) for those ever using e-cigarettes at baseline. More frequent e-cigarette use (at least once per month) at baseline was relatively rare, although more frequent use did appear to be more strongly associated with the three follow-up smoking measures (table 2).

**Table 2:** Relationships between e-cigarette use at baseline (aged 13–14 years) and smoking cigarettes or any combustible tobacco 24 months later (aged 15–16 years) among those who were never smokers at baseline (n=3994)

Smoking at age 15–16 years	Baseline e-cigarette use			
	Never	Tried	Infrequent	Frequent
		(1–2 times)	(1/month–1/week)	(>1/week)
	**n (%**)	**n (%**)	**n (%**)	**n (%**)
Cigarette smoking				
Never	2743 (68.7)	405 (10.1)	30 (0.8)	2 (0.1)
Once	285 (7.2)	147 (3.7)	11 (0.3)	1 (0.0)
Used to smoke	89 (2.2)	63 (1.6)	8 (0.2)	5 (0.1)
Rarely (<1/week)	72 (1.8)	44 (1.1)	5 (0.1)	1 (0.0)
Occasional (1–6/week)	21 (0.5)	18 (0.5)	1 (0.0)	0 (0.0)
Frequent (>6/week)	25 (0.6)	12 (0.3)	6 (0.2)	0 (0.0)
Any recent tobacco smoking				
None	3010 (75.4)	556 (13.9)	37 (0.9)	4 (0.1)
One or more days	226 (5.7)	133 (3.3)	23 (0.6)	5 (0.1)

### Prospective analyses

Ever smoked cigarettes at follow-up (table 3, left-hand panel) was significantly predicted by baseline ever used e-cigarettes (model 1; OR=4.03, p<0.001). It was attenuated but remained significant when controlling for covariates (model 2; OR=2.78, p<0.001). Ever smoked cigarettes was significantly higher in adolescents who were ever users of e-cigarettes, female, non-white, had friends who smoked, more impulsive, had more positive attitudes and perceived behavioural control about smoking and in the control condition. There was also a significant interaction between ever used e-cigarettes and friends smoking (model 2; OR=0.72, p<0.05). Decomposition of this interaction indicated that the impact of ever used e-cigarettes on subsequent ever smoking initiation was greater in those with no friends who smoked (OR=4.23, 95% CI 3.19 to 5.61, p<0.001) compared with those with one or more friends who smoked (OR=2.58, 95% CI 2.05 to 3.24, p<0.001). Analyses (model 3) revealed that condition significantly moderated the impact of e-cigarette smoking (OR=0.50, 95% CI 0.33 to 0.74, p<0.001) and the interaction between ever used e-cigarettes and friends’ smoking (OR=1.70, 95% CI 1.01 to 2.88, p<0.05). Decomposition of the moderation effect for condition indicated that the impact of ever used e-cigarettes on likelihood of ever smoked cigarettes was attenuated in the intervention (OR=1.83, 95% CI 1.35 to 2.48, p<0.001) compared with the control (OR=4.53, 95% CI 3.41 to 6.03, p<0.001) condition. The interaction between ever used e-cigarettes and friends’ smoking became non-significant in the intervention condition (OR=0.99, 95% CI 0.66 to 1.47, p>0.05) but remained significant in the control condition (OR=0.46, 95% CI 0.33 to 0.64, p<0.001), reflecting the fact that ever used e-cigarettes had a stronger effect among those with no friends who smoked.

**Table 3:** Association of ever used cigarettes (left-hand panel), any recent tobacco smoking (middle panel) or regular smoker (right-hand panel) at follow-up with predictors among never users of combustible cigarettes at baseline (over 24 months; n=3994)

Predictors	Ever smoked cigarettes		Any recent tobacco smoking		Regularly smoked cigarettes	
	OR (95% CI)	P value	OR (95% CI)	P value	OR (95% CI)	P value
Model 1 without covariates						
Never used e-cigarettes	1.00		1.00		1.00	
Ever used e-cigarettes	4.03 (3.33 to 4.88)	<0.001	3.38 (2.72 to 4.21)	<0.001	3.60 (2.35 to 5.51)	<0.001
Model 2 with covariates						
Never used e-cigarettes	1.00		1.00		1.00	
Ever used e-cigarettes	2.78 (2.20 to 3.51)	<0.001	2.17 (1.76 to 2.69)	<0.001	1.27 (1.17 to 1.39)	<0.001
Male	1.00		1.00		1.00	
Female	1.41 (1.29 to 1.54)	<0.001	1.16 (1.08 to 1.25)	<0.001	1.08 (1.04 to 1.12)	<0.001
Ethnicity=non-white	1.00		1.00		1.00	
Ethnicity=white	0.81 (0.68 to 0.98	<0.05	0.59 (0.50 to 0.70)	<0.001	0.91 (0.85 to 0.98)	<0.05
Low family affluence	1.00		1.00		1.00	
High family affluence	0.92 (0.83 to 1.03)	>0.05	0.89 (0.80 to 0.98)	<0.05	0.88 (0.82 to 0.93)	<0.001
Free school meals=low	1.00		1.00		1.00	
Free school meals=high	1.12 (0.93 to 1.35)	>0.05	1.10 (0.97 to 1.24)	>0.05	1.03 (0.96 to 1.10)	>0.05
Friend smokers=none	1.00		1.00		1.00	
Friend smokers=more than none	1.49 (1.28 to 1.72)	<0.001	1.53 (1.33 to 1.76)	< 0.001	1.03 (0.97 to 1.10)	>0.05
Family smokers=none	1.00		1.00		1.00	
Family smokers=one or more	1.10 (0.99 to 1.22)	>0.05	1.07 (0.99 to 1.17)	>0.05	0.95 (0.91 to 1.00)	>0.05
Impulsivity	1.30 (1.26 to 1.35)	<0.001	1.23 (1.19 to 1.26)	<0.001	1.03 (1.01 to 1.05)	<0.01
Intentions=low	1.00		1.00		1.00	
Intentions=high	1.58 (1.24 to 2.02)	<0.001	1.24 (1.00 to 1.54)	>0.05	1.12 (0.98 to 1.29)	>0.05
Attitude=low	1.00		1.00		1.00	
Attitude=high	1.18 (1.05 to 1.33)	<0.01	1.21 (1.04 to 1.41)	<0.05	0.98 (0.92 to 1.06)	>0.05
Perceived norms=low	1.00		1.00		1.00	
Perceived norms=high	1.02 (0.88 to 1.19)	>0.05	1.11 (0.93 to 1.33)	>0.05	1.15 (1.06 to 1.24)	<0.01
Perceived behavioural control=low	1.00		1.00		1.00	
Perceived behavioural control=high	1.18 (1.02 to 1.37)	<0.05	>0.05 (0.94 to 1.18)	>0.05	1.08 (1.02 to 1.15)	<0.01
Self-efficacy=low	1.00		1.00		1.00	
Self-efficacy=high	1.15 (0.98 to 1.35)	>0.05	1.19 (0.89 to 1.40)	>0.05	1.01 (0.93 to 1.10)	>0.05
Friends smoking × e-cigarette use	0.74 (0.62 to 0.88)	<0.05	0.70 (0.53 to 0.93)	<0.01	0.90 (0.81 to 1.01)	>0.05
Control condition	1.00		1.00		1.00	
Intervention condition	0.72 (0.61 to 0.86)	<0.001	0.86 (0.76 to 0.96)	<0.05	0.99 (0.93 to 1.05)	>0.05

Ever smoked cigarettes: model 1 without covariates, −2 log-likelihood function = −5585.3; model 2 with covariates, −2 log-likelihood function = −4915.4; any recent tobacco smoking: model 1 without covariates, −2 log-likelihood function = −5564.8; model 2 with covariates, −2 log-likelihood function = −4578.6; regularly smoked cigarettes: model 1 without covariates, −2 log-likelihood function = −5473.6; model 2 with covariates, −2 log-likelihood function = −4076.6.

In relation to any recent tobacco smoking at follow-up (table 3, middle panel), smoking was significantly predicted by baseline ever used e-cigarettes (model 1; OR=3.38, p<0.001) and attenuated but remained significant when controlling for covariates (model 2; OR=2.17, p<0.001). Recent smoking was significantly higher in adolescents who at baseline were ever users of e-cigarettes, female, non-white, had lower family affluence, had friends who smoked, more impulsive, had more positive attitudes about smoking and in the control condition. There was also a significant interaction between ever used e-cigarettes and friends smoking (model 2; OR=0.70, p<0.01). Decomposition of this interaction indicated that the impact of ever used e-cigarettes on subsequent any recent tobacco smoking was greater in those with no friends who smoked (OR=3.96, 95% CI 2.94 to 5.33, p<0.001) compared with those with one or more friends who smoked (OR=2.07, 95% CI 1.58, to 2.72, p<0.001). Analyses (model 3) did not reveal any significant moderating effects of condition on relationships between predictors and recent smoking.

Finally, in relation to regularly smoked cigarettes at follow-up (table 3, right-hand panel), smoking was significantly predicted by baseline ever used e-cigarettes (model 1; OR=3.60, p<0.001) and was attenuated but remained significant when controlling for covariates (model 2; OR=1.27, p<0.001). Regularly smoked cigarettes was significantly higher in adolescents who were ever users of e-cigarettes, female, non-white, had low family affluence, more impulsive, had more positive norms and perceived behavioural control about smoking. There was no significant interaction between ever used e-cigarettes and friends smoking on regular smoking (model 2; OR=0.90, p>0.05). Analyses (model 3) did not reveal any significant moderating effects of condition on relationships between predictors and regularly smoked cigarettes.

## Discussion

The present research showed that never smoking 14-year olds who ever versus never used e-cigarettes were more likely to report having smoked at least once or more regularly 24 months later. These effects remained (although attenuated) when controlling for various predictors of smoking. The degree of attenuation was greater for regular smoking than for other smoking measures. This study adds to the growing number of US^12–15^ and UK^16 17^ studies showing that e-cigarette use in adolescents is reliably associated with subsequent smoking. The current results are comparable to those reported in a recent meta-analysis of nine such studies (OR=3.50, 95% CI 2.38 to 5.16) for ever smoking based on comparing never versus ever users of e-cigarettes.^30^ The reviewed studies were generally over a period of 12 months (five of nine studies) with a maximum follow-up period of 18 months and focused on ever smoking. In contrast, the present study was over 24 months and showed effects for ever smoking, any recent tobacco smoking and regular cigarette smoking (see^15^ for similar results). Together, these studies suggest that it is unlikely that the high rates of dual use of e-cigarettes and smoking observed in the US^5–8^ and UK^9–11^ in cross-sectional surveys of adolescents are entirely attributable to cigarette users subsequently taking up e-cigarettes. In our sample, at follow-up approximately one-third (177/585=30.3%) of those who used both e-cigarettes and cigarettes reported using e-cigarettes first (a further 191/585=32.6% reported using cigarettes first, and the remainder 217/585=37.1% could not remember which they tried first).

Our findings also indicated that the association between ever use of e-cigarettes and subsequent ever smoked cigarettes or any recent tobacco smoking (but not regularly smoked cigarettes) was significantly stronger among adolescents with no friends who smoked, a group usually considered to be less susceptible to smoking initiation. This replicates and extends previous research^17^ and extends our previous findings^16^ to a larger sample over a longer time period. Barrington-Trimis *et al* 31 reported similar moderation effects for intentions to smoke. This appears inconsistent with the idea that e-cigarette users are more interested in all forms of nicotine use and the fact that e-cigarette use came first is purely coincidental.^32^ Nevertheless, the fact that adolescent smoking is decreasing at the same time as e-cigarette use is increasing^5–11 33^ appears inconsistent with a causal link between e-cigarette use and subsequent smoking.

Importantly, our research showed for the first time that an intervention^18 19 21 22^ designed to reduce smoking initiation in adolescents significantly weakened the impact of ever used e-cigarettes on follow-up ever smoked cigarettes (but not any recent tobacco smoking or regularly smoked cigarettes). This is the first study to investigate the impact of an antismoking intervention on this relationship. Although the ever used e-cigarettes to ever smoked relationship was attenuated in the intervention condition, it remained significant. This relationship was in addition to a main effect of the intervention on lower rates of ever smoked at follow-up. It is also worth noting that the intervention did not cover e-cigarettes or relationships between e-cigarettes and subsequent smoking.^18 19^ A more targeted intervention may arguably have been more successful in reducing the ever used e-cigarettes to subsequent smoking association to zero.

Like other similar studies, our research provides only limited insights into the mechanism relating ever use of e-cigarettes to subsequent smoking. This means we need to remain cautious in making policy recommendations based on these findings. Since the start of our work, UK legislation has banned marketing and selling e-cigarettes to minors and UK agencies are required to enforce age of sale, child and tamper proof packaging, display age of sale signage and health warnings on e-cigarette packaging.^34^ Nevertheless, our findings emphasise the value of regulating the marketing/sale of e-cigarettes to minors in countries where such measures are not in place, particularly given that e-cigarette advertising has been shown to reduce perceived harm of occasional cigarette use.^35^

Our study has a number of strengths including a large demographically diverse sample, assessment of effects over 24 months, exploration of effects on ever smoked cigarettes, any recent tobacco smoking and regularly smoked cigarettes, validated self-reported smoking measures, exploration of covariates and particularly the assessment of the impact of an antismoking intervention. There are also weaknesses. First, our self-reported measure of e-cigarette use was not validated against objective measures. Second, we did not distinguish types of e-cigarette use (eg, delivery method, nicotine content). Furthermore, our study is restricted to first-generation e-cigarette devices, which less closely mimic combustible cigarette in their nicotine delivery profile.^36^ Exploring relationships between the use of new generations of e-cigarettes both containing nicotine or not and different flavourings and subsequent smoking is an important issue for further research. Although e-cigarettes with higher levels of nicotine seen in the US are currently prohibited in the UK, a recent report indicated that rechargeable devices with a tank that users can refill with liquid are now the most widely used among 11–18-year olds, with fruit-flavoured liquid being the most popular.^9^ Third, our analyses were restricted to ever use of e-cigarettes due to low rates of regular e-cigarette use at baseline (table 2). Relatedly, there were only 81 regular smokers in our sample at follow-up restricting the power of analyses on regular smoking.

In summary, this is the first study to report the impact of an antismoking intervention on the relationship between ever use of e-cigarettes and subsequent smoking 24 months later among UK adolescents. Despite including a range of covariates,^12–17^ it is possible that other third variables (eg, sensation seeking) could have been responsible for the observed relationships. Therefore, while acknowledging that a causal relationship may be plausible, we cannot confirm this based on our findings and the trends observed over the same time period in the UK are inconsistent with such a causal relationship. Future research could seek to disentangle these apparently contrary findings and assess dose–response relationships between e-cigarette and subsequent smoking over different time periods in broader age ranges of adolescents while controlling for a range of covariates.

**What this paper adds**

- Previous research: A growing number of studies (mainly among US and UK adolescents) indicate that self-reported e-cigarette use is associated with subsequent smoking initiation. However, in general, these studies were conducted over relatively short time periods (12 months), focused only on smoking initiation and not on more regular smoking, did not validate their self-reported smoking measures against objective measures and did not examine the impact of smoking prevention interventions.
- Interpretation: The present research replicates previous findings in this area in showing a significant association between e-cigarette use and subsequent smoking initiation. It also shows similar effects for measures of regular smoking. These relationships were observed over a period of 24 months in measures of ever smoked cigarettes, any recent tobacco smoking and regularly smoked cigarettes. The strength of these associations was reduced but remained significant when controlling for various predictors of smoking. Importantly, the present research showed for the first time that an intervention to reduce smoking initiation attenuated the relationship between e-cigarette use and ever smoked cigarettes (although it remained significant). This suggests the value of interventions to reduce smoking initiation even in groups of adolescents who try e-cigarettes first. Similar to recent UK studies, the present data also showed that the relationship between e-cigarette use and ever smoked cigarettes or any recent tobacco smoking was stronger in those with no friends who smoked at baseline (a group usually thought to be at low risk of starting smoking) versus some friends who smoked at the initial time point. These latter findings are more consistent with the view that e-cigarette use is a risk factor for smoking initiation than the view that e-cigarette use may simply be a marker for those who would go on to smoke cigarettes even without having tried e-cigarettes. However, it is notable that this moderation effect was not observed for regular smoking.
